# Efficacy of an Optimised Bacteriophage Cocktail to Clear *Clostridium difficile* in a Batch Fermentation Model

**DOI:** 10.3390/antibiotics7010013

**Published:** 2018-02-13

**Authors:** Janet Y. Nale, Tamsin A. Redgwell, Andrew Millard, Martha R. J. Clokie

**Affiliations:** 1 Department of Infection, Immunity and Inflammation, University of Leicester, Leicester LE1 9HN, UK; jn142@le.ac.uk (J.Y.N.); adm39@leicester.ac.uk (A.M.); 2 School of Life Sciences, University of Warwick, Coventry CV4 7AL, UK; T.Redgwell@warwick.ac.uk

**Keywords:** *Clostridium difficile*, *Clostridium difficile* infection, bacteriophages, phage therapy, microbiome, in vitro fermentation model

## Abstract

*Clostridium difficile* infection (CDI) is a major cause of infectious diarrhea. Conventional antibiotics are not universally effective for all ribotypes, and can trigger dysbiosis, resistance and recurrent infection. Thus, novel therapeutics are needed to replace and/or supplement the current antibiotics. Here, we describe the activity of an optimised 4-phage cocktail to clear cultures of a clinical ribotype 014/020 strain in fermentation vessels spiked with combined fecal slurries from four healthy volunteers. After 5 h, we observed ~6-log reductions in *C. difficile* abundance in the prophylaxis regimen and complete *C. difficile* eradication after 24 h following prophylactic or remedial regimens. Viability assays revealed that commensal enterococci, bifidobacteria, lactobacilli, total anaerobes, and enterobacteria were not affected by either regimens, but a ~2-log increase in the enterobacteria, lactobacilli, and total anaerobe abundance was seen in the phage-only-treated vessel compared to other treatments. The impact of the phage treatments on components of the microbiota was further assayed using metagenomic analysis. Together, our data supports the therapeutic application of our optimised phage cocktail to treat CDI. Also, the increase in specific commensals observed in the phage-treated control could prevent further colonisation of *C. difficile*, and thus provide protection from infection being able to establish.

## 1. Introduction

Antimicrobial resistance is a global health threat to clinical practice and public health [1,2,3,4]. It is estimated that the continued rise in multidrug resistance (MDR) will cause 10 million people to die worldwide by 2050 and cost 100 trillion USD [5]. To effectively control bacterial infections, novel effective antimicrobials with target specificity and high efficiency are urgently needed [6,7,8]. Although bacteriophages or phages (viruses which specifically lyse bacteria) were first isolated over 100 years ago, for a long period they were mainly the focus of fundamental research. However, particularly over the last decade, there has been an increasing interest in the isolation, characterisation and development of phages for therapeutic use in humans, animals, and plants [9,10,11,12,13]. This revived interest is mainly driven by problems associated with ineffective antibiotics. These natural bacterial predators have the potential to provide a safe and suitable supplement, or replacement for antibiotics because of their specificity and amplification at the site of infection [14,15,16,17]. Indeed, phage products have been developed for medical use, and some can be found as over-the-counter medicines and are used as decontamination agents in food industries [10,18,19,20].

*Clostridium difficile* is a notorious nosocomial bacterium that remains a major cause of infectious diarrhea, with high morbidity and mortality in the elderly and in immunocompromised patients worldwide [21,22,23,24,25]. *C. difficile* surveillance for the United Kingdom showed that there were 19,269 reported cases of *C. difficile* infection (CDI) and 488 (~3%) fatalities in 2015. In the US, ~500,000 CDI cases are reported annually, with approximate 30,000 deaths, 20% recurrent rates, and an estimated treatment cost of ~$10,000 per case [21,22,26]. CDI is becoming increasingly difficult to treat because of the emergence of severe and antibiotic-resistant ribotypes, and very limited treatment options [6,27,28]. Currently, only three antibiotics are available on the market for CDI treatment. Metronidazole is cheap, largely effective, and is recommended for initial use in moderate or non-severe episodes [29,30,31]. However, there are problems associated with its efficacy to treat some important prevalent and clinically relevant ribotypes, resistance has also been seen towards this antibiotic, and it is associated with health-related complications such as low birth weight [30,32,33]. Vancomycin is the antibiotic of choice for moderate to severe CDI but its use, particularly if long-term, can promote the emergence of vancomycin-resistant enterococci [34,35]. Also reduced susceptibility have been reported in *C. difficile* leading to recurrent infection, thus it is suboptimal [36,37]. Fidaxomicin is a highly specific antibiotic that has been shown to be effective when vancomycin treatment has failed [38] but it is expensive ($3360 compared to $1273 for vancomycin or $21.90 for metronidazole—all per course) and may not be cost-effective for some strain-specific CDIs [39,40,41]. This complex relationship between *C. difficile* and antibiotics is compounded by the fact that they generally have a detrimental impact on the gut microbiota, which leads to dysbiosis that then enables *C. difficile* to colonise the gut and cause disease. Therefore, there is a clear need to develop additional antimicrobials with increased target specificity in order to efficiently remove this pathogen but leave other components of the gut microbiota intact [6,10]. 

Previous reports have described the isolation of phages that specifically target *C. difficile* and demonstrated the use of different in vivo and in vitro models to test the specificity and efficacy of the phages to selectively eradicate this bacterium [42,43,44,45,46]. The commonly used in vivo model for CDI and *C. difficile* phage therapy is the hamster model, which is useful as hamsters demonstrate the classical CDI clinical symptoms seen in humans [47,48]. However, the model is difficult to use because of the exquisite sensitivity of hamsters to *C. difficile* toxins, high costs, and inherent technical issues associated with working with these animals [49,50]. Therefore, alternative models such as the wax moth *Galleria mellonella* larva have been developed as suitable replacement models to probe many aspects of CDI phage therapy [46]. Other models that have been used to study *C. difficile* phage therapy are the in vitro gut and batch fermentation models [44,51]. Although these models have been developed to study the gut microbiome and pharmokinetics of antibiotics, very few studies have applied them to study *C. difficile* phage therapy [38,50,52].

The four myoviruses CDHM1, 2, 5, and 6 used in this study were isolated from the environment and were well characterised in our laboratory [45,53]. This optimised phage cocktail was the first phage mix shown to completely clear *C. difficile* in pure cultures and it was also shown to prevent biofilm formation in vitro. In addition, the phages reduced colonisation in vivo in both hamster and wax moth larva CDI models [45,46]. The data obtained from these models provided novel insights into the therapeutic applications of these phages to treat CDI. However, more information is needed in order to determine the specificity of this phage set to *C. difficile*, and to establish their ability to clear the target pathogen in the presence of competitive pressure from other components of the human gut microbiota. Indeed, no previous publications have examined the potential impact of the application of *C. difficile* phages on the human microbiome. Therefore, we developed and present results from an in vitro phage therapy assay using a batch fermentation model. To do this, we obtained human feces from a specific age profile of healthy volunteers (with full ethical consent) in order to examine the impacts of our phage set on a wide range of human microbiota. The work was designed to: (i) determine the efficacy of our optimised phage cocktail to clear a clinically relevant ribotype 014/020 strain in the presence of the gut microbiota, (ii) test the efficiency of the phages using prophylactic or remedial regimens in the targeted eradication of the bacterium in the batch fermentation model, and (iii) ascertain the potential synergistic or antagonistic effect of phage application on culturable and unculturable components of the human gut microbiome. 

## 2. Results

### 2.1. Individual Donors Have a Unique Microbiome Composition

**Figure 1:**
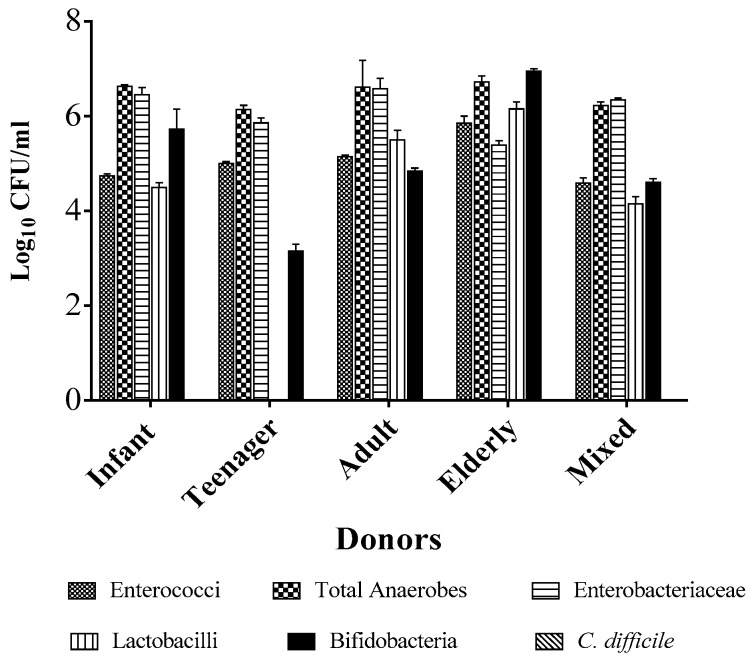
Contributory culturable bacterial counts from each of the individual donors and final cumulative counts of each bacterium added to the fermentation vessels. The bacteria present in the fecal sample of each donor were determined by recovery on selective medium for each bacterial grouping, after which, the samples were mixed together in relatively equal amounts and used to prime the fermentation vessels. The data was analysed using GraphPad Prism 7. Error bars are SEMs of three biological replicates.

To determine the specificity and efficacy of the phages to clear *C. difficile* in the presence of competitive pressure from representative human gut microbiota, we spiked five fermentation vessels containing a minimal medium with combined fecal slurries obtained from four healthy volunteers [54,55,56]. The donors were comprised of individuals from diverse ethnic and age groups (a 70-year-old white British woman, 44-year-old black woman, 17-year-old black girl, and 7-year-old white British boy) to capture a wide range of human gut microbial diversity. Prior to mixing the fecal slurries together, we determined the microbiome composition from the individual donors by resuspending the fecal matter in the minimal medium and enumerating the bacteria present on selective agar media targeting five commonly occurring gut commensals [44,51]. We observed that approximately 10^5^–10^6^ CFU/mL (colony-forming unit per milliliter) of enterococci counts were detected from all the four donors. Similar counts were observed with the lactobacilli group, except that the abundance of this bacterium was very low, hence it was undetectable in the teenager. Relatively higher counts were observed in the total anaerobes and enterobacteria, which ranged from 10^6^ to 10^7^ CFU/mL in all the donors. The bifidobacterial counts were quite variable, from very low counts of ~10^3^ CFU/mL in the teenager, to 10^5^ and 10^6^ CFU/mL in the infant and adult, respectively, and 10^7^ CFU/mL in the elderly donor lady. Interestingly, but not unexpectedly, we did not recover *C. difficile* from any of the donors. When the fecal matter was mixed together and assayed, it was observed that the total anaerobes and enterobacterial numbers were the highest with ~10^6^ CFU/mL, but ~10^5^ CFU/mL was being contributed by the enterococci, lactobacilli, and bifidobacteria (Figure 1).

### 2.2. Phages Cleared C. difficile in the Batch Fermentation Model 

**Figure 2:**
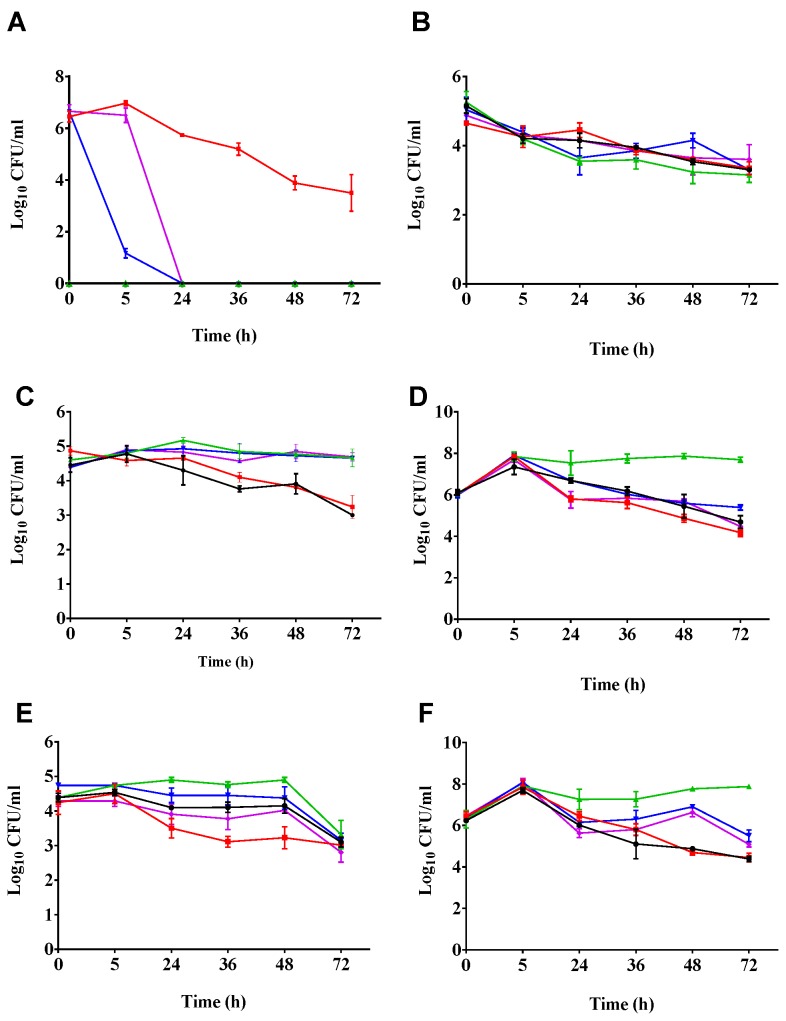
Impact of phage treatment on *C. difficile* and other components of the gut microbiota. The impact of phage treatment was ascertained by recovering the bacteria on selective media for (**A**) *C. difficile*; (**B**) bifidobacteria; (**C**) enterococci; (**D**) enterobacteria; (**E**) lactobacilli; (**F**) total anaerobes*.* The bacterial counts of the different treatment vessels and time points are presented. Black lines, vessel 1, untreated slurries; red lines, vessel 2, *C. difficile* control; green lines, vessel 3, phage-only-treated control; blue lines, vessel 4, prophylaxis regimen, and purple lines, vessel 5, remedial regimen. The data was analysed using GraphPad Prism 7. Error bars are SEMs of 3 biological replicates.

We determined the ability of the phage cocktail to clear CD105LC2 (ribotype 014/020 clinical strain) in the batch fermentation model using two treatments. In the prophylactic regimen, the fecal slurries were exposed to a dose of the phage cocktail followed by a mixture of the phages and bacteria, and subsequently by two doses of the phage cocktail (Table 1). In the first 5 h following bacteria and phage exposure, we observed a ~6-log reduction of CFU/mL of *C. difficile* counts in the prophylactic regimen compared to the bacterial control. When the fermentation vessels were treated remedially, the phage cocktail was added after culturing the bacteria for 5 h. At the 24 h time point (19 h post-phage treatment in the remedial regimen), *C. difficile* was eradicated in both the prophylactic and remedial treatment vessels, and this observation remained consistent from this time until the end of the experiment (72 h). As expected, *C. difficile* was not detected in the untreated (vessel 1) and the phage (vessel 3) controls. However, in the *C. difficile*-only control (vessel 2), we observed that after 5 h of incubation, *C. difficile* numbers began to drop from ~10^7^ CFU/mL (at 5 h) to ~10^4^ CFU/mL at 36, 48, and 72 h, respectively (Figure 2A).

### 2.3. Impact of Phage Treatmens on the Viability of other Components of the Culturable Gut Microbiota

After establishing the efficacy of the phage cocktail to clear *C. difficile* in the batch fermentation model, we investigated their impact on five common major bacterial groups in the human gut. We did this by conducting viability assays on selective media for bifidobacteria, enterococci, enterobacteria, lactobacilli, and total anaerobes in the five fermentation vessels at all the time points examined [44,51].

Bifidobacterial numbers were relatively constant in both the treatment regimens and the three controls throughout the experiment. The ~10^5^ CFU/mL of bacteria observed at 0 h decreased to ~10^4^ CFU/mL in all the treatment regimens as well as in the controls at 5 h. The bacterial numbers remained relatively stable at this level until the end of the experiment (72 h). There was no significant difference in the number of bacteria left in all the treatment vessels at the end of the 72 h time period of the experiment (*p* = 0.05) (Figure 2B).

The enterococci abundance showed distinct changes depending on the treatment. Relatively equal numbers, ~10^5^ CFU/mL of bacteria, were observed at the beginning of the experiment (0 h) in all the treatment vessels, and this number remained consistent until 24 h. After this time, the bacterial numbers dropped to ~10^4^ CFU/mL in vessels 1 and 2, which corresponded to the untreated and *C. difficile* controls, respectively, both not treated with the phages. The numbers for this group of bacteria continued to drop in vessels 1 and 2, and after 72 h, only ~10^3^ CFU/mL of bacteria were recovered. However, in all the phage-treated vessels comprising the phage control (vessel 3), the prophylactic (vessel 4), and the remedial regimens (vessel 5), the enterococci detected remained relatively stable at ~10^5^ CFU/mL from 5 h to 72 h (Figure 2C).

For the enterobacteria, the numbers increased from ~10^6^ to 10^8^ CFU/mL within the first 5 h of the experiment in all the fermentation vessels. After 24 h, the bacterial numbers remained stable in the phage-only treated control, but lower numbers (10^7^ CFU/mL) were observed in the untreated vessel (vessel 1) and the prophylaxis vessel (vessel 3). The remedial and the bacterial control vessels had even lower numbers (10^6^ CFU/mL). After 24 h, higher enterobacterial numbers (~10^8^ CFU/mL) were seen in the phage-only treated vessel (vessel 3), which remained stable throughout the experiment. In the other vessels (vessels 1, 2, 4, and 5), however, the bacterial counts remained lower (at ~10^6^ CFU/mL) than in the phage-treated vessel at 24 h until the experiment was terminated. At the end of the experiment, ~2-log CFU/mL higher numbers of enterobacteria were observed in the phage-only treated vessel (vessel 3), compared to the other vessels (Figure 2D).

We also assayed for lactobacilli counts in all the treatment vessels over the time points. We observed that equal numbers (~10^5^ CFU/mL) of the bacteria were detected in the beginning of the experiment at 5 h in all the vessels. At the 24 h time point, the phage-only treated and the prophylactic-treated vessels had higher bacterial numbers than the remedial regimen, the *C. difficile*, and the untreated control vessels. In all the vessels, the bacterial numbers remained stable at this level and at the subsequent three time points of 24, 36, and 48 h, but steadily declined to ~10^3^ CFU/mL at 72 h (Figure 2E).

The final bacterial group assayed on selective media was the total anaerobes. As observed for the enterococci, the total number of anaerobes increased markedly from ~10^6^ CFU/mL at 0 h to 10^8^ CFU/mL at 5 h in all the vessels. However, the total number of anaerobes (~10^7^ CFU/mL) in the phage-only treated control vessel (vessel 3) was ~1-log CFU/mL higher than in the other four treatment vessels (vessels 1, 2, 4, and 5) at 24 and 36 h. At 48 h however, the total anaerobe count was higher in the phage-only treatment (vessel 3), with ~10^8^ CFU/mL recovered, followed by the prophylaxis and remedial regimen vessels with ~10^6^ CFU/mL of bacteria detected. The control untreated slurries (vessel 1) and the *C. difficile* control (vessel 2) had relatively lower numbers (~10^5^ CFU/mL) at 72 h (Figure 2F).

### 2.4. Metagenomics Analysis of the Impact of Phage Treatment on the Total Microbiome within the Gut Fermentation Vessels

**Figure 3:**
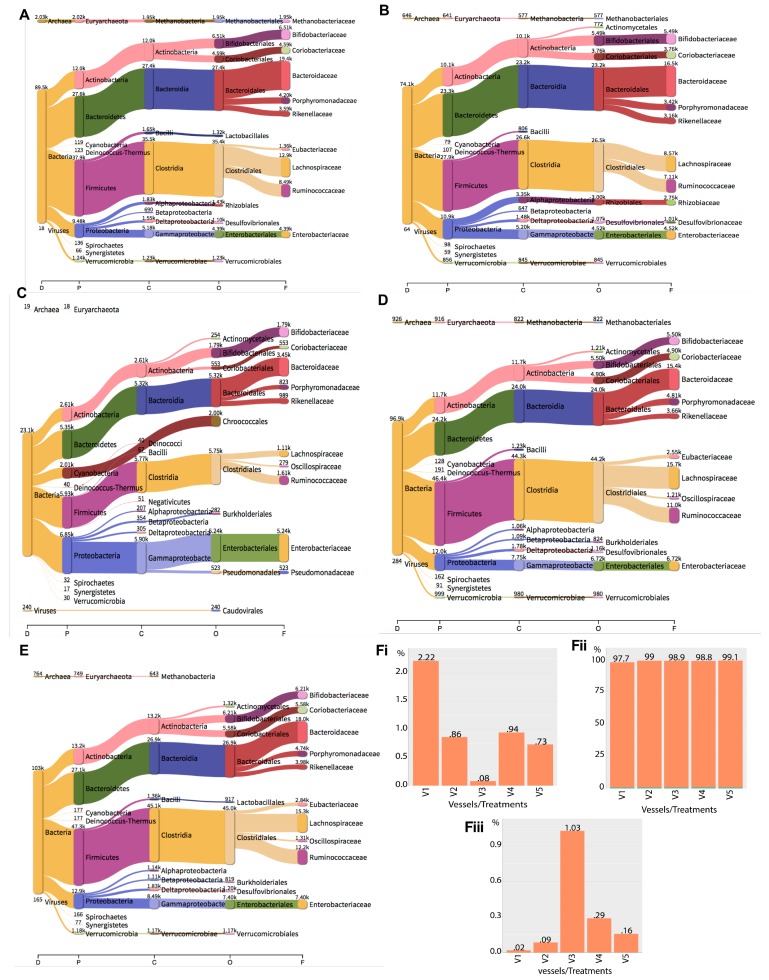
Analysis of the 10 most abundant taxa from Archea, Bacteria, and Viruses as ascertained by the metagenomics data. Total genomic DNA was extracted at 24 h time point from (**A**) vessel 1, untreated slurries; (**B**) vessel 2, *C. difficile* control; (**C**) vessel 3, phage-only-treated control; (**D**) vessel 4, prophylaxis regimen; (**E**) vessel 5, remedial regimen. The samples were prepared using NexteraXT sample preparation kit and sequenced on MiSeq platform using V2 (2 × 250 bp) chemistry. The resulting fastq files were trimmed with Sickle, and the metagenomes were assembled using megahit. An overview of the 10 most abundant taxa: Phyla (P), Classes (C), order (O), and family (F) are shown for each treatment vessel, as visualised using Pavian. The percent reads mapped to Archaea, Bacteria, and Viruses in the vessels at 24 h are shown in (**Fi**), (**Fii**), and (**Fiii**), respectively.

The viability assays confirmed a complete eradication of *C. difficile* at the 24 h after phage treatment in both the prophylactic and remedial fermentation vessels. The beginning of an effect on five other bacterial groups was also observed. To probe these observations more deeply and to determine the impact on the components of the microbiota, including those that cannot be cultured, the total DNA from the five treatment vessels at the 24 h time point was extracted and used as a template for whole metagenomics analysis. The total reads per vessel at 24 h were mapped to relevant sequences representing all three domains of life (Table 2). The percentage of reads for each domain was normalised against the total number of reads found in each vessel. The highest percentage of reads for bacteria was found in vessel 5, with 99.11%, whereas the lowest percentage of reads was found in vessel 1, with 97.76% reads. We observed that Firmicutes, Bacteroidetes, Proteobacteria, Actinobacteria, Cyanobacteria, Euryarchaeota, Verrucomicrobia, Deinococcus-Thermus, Spirochaetes and Synergistetes abundances were consistently the most abundant among the bacterial phyla examined, irrespective of the treatment vessel (Table S1, Figure 3A–D,Fi). Although the individual groups of bacteria remained consistent in all treatments, their abundances varied considerably in the vessels. We observed that percent reads mapped to Actinobacteria were higher in the none-phage-treated vessels (vessel 1, 26.2% and vessel 2, 26.9%) compared to vessels 3 (22.4%), 4 (23.8%), and 5 (25.3), which corresponded to phage-treated vessels (Table S1, Figure 3A–E). This pattern in all the vessels was also observed for the Bacteroidetes, for which the reads found in non-phage-treated vessels were higher compared to those in phage-treated vessels (Table S1). In contrast, the Deinococcus levels were higher in vessels 3, 4, and 5, which contained the phages, but reduced in non-phage-treated vessels (vessels 1 and 2). The Firmicutes and Verrucomicrobia had reduced abundance in the phage-only-treated slurries compared to the other four vessels (Table S1). Conversely, the Cyanobacteria, Enterobacteriaceae, and Proteobacteria abundance was elevated at 24 h in the phage-only-treated control vessel compared to the other four vessels. The abundance of the Spirochaetes in the prophylaxis treatment regimens was comparable to the level found in the untreated slurries (vessel 1). Consistent with our viability assays, we observed that Bifidobacteriaceae, Enterobacteriaceae, Lactobacillales as well as the Coriobacteriaceae, Bacteroidaceae, Porphyromonadaceae, Rikenellaceae, Eubacteriaceae, Lachnospiraceae, Rhizobiales, Desulfovibrionales and Ruminococcacea abundances were considerably high in vessel 3 from the metagenomics data (Figure 3A–D,Fi).

The percentage of reads mapped to Archaea was found to be highest in the untreated vessel (vessel 1), with 2.2% reads mapped to this domain in this vessel at the 24 h time point. The remaining vessels, V2–5, had low percent reads (0.08–0.94) mapped to Archaea compared to the untreated vessel 1. The phylum Euryarchaeota, consisting of the family Methanobacteriaceae was consistently found in all the vessels, although its abundance was considerably lower in the phage-only-treated vessel (vessel 3) (Figure 3Fii). 

Consistent with the other two phyla, we found reads corresponding to viruses to be represented in all the treatment vessels. For the percentages of reads which mapped to the viruses, we found the highest level in vessel 3 (phage-only-treated vessel) with 1.029% of the total reads, and lower levels in all other treatment vessels (vessel 1, 2, 4, and 5). Vessel 1 had the lowest percent viral reads (0.0196) compared to vessels 2, 3, 4, and 5, which had 0.08553, 1.029, 0.2895, and 0.1581%, respectively (Figure 3Fiii). 

## 3. Discussion

The need for alternative therapeutics to combat antibiotic resistance is clear [5,57]. The challenges posed by MDR are current and serious and so cannot be ignored. Consequently, significant resources are being channeled towards understanding the root causes of MDR and developing effective approaches to tackle the associated health threats [8,58,59]. There is an urgent clinical unmet need for novel treatments for *C. difficile*, the causal agent of CDI [6,60]. Recent reviews on past, current, and future options for CDI treatment concluded that phage therapy has significant potential as a treatment because of its specificity, amplification at infection sites, and minimal deleterious impact to the gut microbiome [6,16,43]. The development of phage treatments for this pathogen has been hampered by the lack of strictly virulent phages. Furthermore, although past data has demonstrated the efficacy of *C. difficile* phages to clear the bacterium in vitro and in vivo, there is the lack of preclinical data to ascertain the impact of these phages on the human gut microbiome [45,46]. Previously, we developed the first effective *C. difficile* phage cocktail, which consists of four well-characterised, broad host range myoviruses [45,46]. In this study, we showed as a proof of concept that the cocktail can effectively clear a clinically prevalent *C. difficile* ribotype isolate in a batch fermentation model, and that their application promotes the growth of other human gut commensals. 

The two previous reports on *C. difficile* phage therapy using in vitro gut models used a batch fermentation model over a 48 h time period [51] or a three-component continuous colon model over a 35 day period [44]. In both assays, the effect of one phage, ΦCD27, to clear NCTC11204, a ribotype 001 strain, was investigated. The two reports are consistent with our observation that the prophylactic regimen is more effective at clearing *C. difficile* than the remedial regimen. Although both previous data sets showed a significant reduction (~6-log CFU/mL) in the prophylaxis treatment, *C. difficile* (~1-log CFU/mL) was still detected in the 48 h report [51], and up to 10^8^ CFU/mL of cells or spores were detected in the 35 day report at the end of the assays [44]. Whilst ΦCD27 was shown to be active in the prophylactic regimen, the regrowth observed could be attributed either to resistance-developing, an inherent insensitivity of the bacterium to the phages, or the generation of resistant lysogenic clones, as shown when the bacterium was treated with the phage at a multiplicity of infection (MOI) of 7 [51]. Since all published *C. difficile* phages are temperate and encode an integrase gene, which mediates their integration into host genomes, the development of lysogenic mutants using a single phage for therapy occurs, as shown in previous reports [45,47,51]. In our previous work and in work presented here, we have demonstrated that the impacts of lysogeny and/or phage or antibiotic resistance are mitigated by the application of an optimised diverse phage cocktail [45,46]. *C. difficile* was fully lysed regardless of whether a prophylactic or remedial regimen was applied, and remained undetectable till the end of the experiment (72 h), as shown in this study (Figure 2A) and in our previous in vitro models [45,46]. The phages used here exhibit a complementary effect, whereby resistant or lysogenic clones produced by one phage or antibiotic treatment become susceptible to infection by another phage in the mix, leading to complete eradication of *C. difficile* in vitro and significant reduction of colonisation in vivo [45,46]. 

The advantages of using a phage cocktail was also demonstrated in our remedial regimen, where we observed a ~7-log CFU/mL reduction in *C. difficile* counts, and the bacterium was completely eliminated from vessel 5 (remedial regimen) within 24 h of the post-phage treatment without any detectable regrowth (Figure 2A). In contrast, there was only a >1-log CFU/mL reduction when the single phage ΦCD27 was applied remedially at an MOI of 10 at this time and at subsequent time points, and no observable impact was reported when the phage was applied at an MOI of 7 [51]. The lower remedial impact of *C. difficile* phages was also observed in other in vitro assays [45,46,47,48]. Obviously, these data further support the fact that phage therapy for CDI will require the application of optimised phage combinations, as previously reported for treating other bacterial species [13,61]. Although our phage cocktail was optimised for the ribotype 014/020, work is currently ongoing in our laboratory to determine suitable phage combinations for other prevalent and severe ribotypes.

In our fermentation model, we have for the first time studied the impact of phages on microbiomes derived from fecal samples from four healthy volunteers from different age groups and ethnicities to prime the fermentation vessels. Although we used fecal matter from healthy live volunteers from different age groups, other work used human fecal matter of deceased individuals [62] or healthy elderly individuals [44,51,52,56]. Whilst the elderly group reflects the majority of people commonly predisposed to CDI because of their weak immune system, other age groups are also susceptible to the pathogen, though to a lesser extent [63,64]. In addition, human gut microbiomes have been shown to vary greatly and be shaped by individual lifestyles, age groups, and geographical regions, and have been studied in combined emulsions (Figure 1) [54,55,65]. 

Although *C. difficile* is often a natural human commensal, we did not recover it from the fecal samples of our donors using our viability assays. *C. difficile* is generally considered to be an opportunistic bacterium and could remain in the gut of a healthy individual without causing disease until there is a disruption of the microbial balance (dysbiosis) through antibiotic use, triggering *C. difficile* to colonise the gut and causing disease [6,66,67]. *C. difficile* could possibly be present in the guts of our donors but in low abundance, hence it could not be detected in the feces [67]. In addition, the decline in *C. difficile* counts, as observed in the bacterial control after 24 h, has been reported previously and could reflect the response of the bacterium outside the natural gut environment or the depletion of nutrients in the medium used [44,51]. 

Our observations from the viability assays and metagenomic analysis show that both the prophylactic or remedial phage regimens did not have a significant detrimental impact on the five bacterial groups examined and concur with other previous phage therapy assays [44,51]. Similarly, we did not observe a huge difference in the abundance of bacteria in the phage-treated vessels and controls in the metagenomics data, and this clearly supports the advantages of phage therapy over antibiotics [68]. Because of their specificity, phages are able to infect the targeted bacteria, preserving the commensal niche as opposed to chemical antibiotics, which have a broader activity and may induce superinfection by some species such as *C. difficile* [15,69].

Prior to our work, no data had examined the impact of just *C. difficile* phages on other components of the microbiota during therapy. The two previously published fermentation model reports examined the impact of phages on *C. difficile* clearance and the corresponding impact on other commensals but did not examine the effect of phages alone on the gut microbiota [44,51]. Here, we showed that the abundances of certain gut commensals were elevated and restored to the initial levels of the donor samples during phage administration, and this clearly links to the high viral abundance found in vessel 3 (Table 2). This observation strongly suggests that the phages could promote the growth of natural human bacteria, providing health benefits, and thus could protect the gut from *C. difficile* colonisation [69]. The possible roles of phages in the restoration of the gut microbiomes of CDI patients have previously been reported and may provide biological insights into the mechanisms of fecal transplantation. For example, previous data showed that fecal matter containing higher diversity of Caudovirales led to increased richness, diversity, and evenness of these viral particles when transplanted to CDI patients. A concomitant increase in the abundance of other gut commensals (such as Proteobacteria and Actinobacteria) and the resultant resolution of CDI were also observed in the majority of the recipient patients [70]. Similarly, the administration of fecal filtrates from healthy humans via nasojejunal tubes restored the normal stool habits and eliminated CDI symptoms in five symptomatic chronic relapsing CDI patients in another study [71]. 

## 4. Materials and Methods

### 4.1. Bacterial Isolates and Phage Cocktail Used in This Study

In this study, two *C. difficile* isolates were examined. The first, CD105HE1, is an environmental isolate of ribotype 076 and was used as the propagating host for the phages [53]. The second bacterial isolate, CD105LC2, was the test strain for the gut fermentation model and belonged to the clinically prevalent ribotype 014/020 [45]. The bacterial isolates were routinely cultured on brain heart infusion (BHI) agar (Oxoid, Hampshire, UK) supplemented with 7% defibrinated horse blood (Thermo Scientific, Hampshire, UK) for 48 h prior to use, or stored in cryogenic storage tubes (Abtek Biologicals Ltd., Liverpool, UK) at −80 °C. The bacterial culture used for the gut fermentation experiments was produced by inoculating a single colony of the test bacterium in 5 mL of pre-reduced fastidious anaerobic broth (BioConnections, Knypersley, UK) and incubating anaerobically (10% H_2_, 5% CO_2_ and 85% N_2_, Don Whitley Scientific, West Yorkshire, UK) at 37 °C for 18–24 h. A 1:100 dilution of the overnight culture was prepared in 10 mL BHI broth, incubated until OD_550_~0.25–0.3 (~10^8^ CFU/mL) was attained, and used to inoculate the fermentation vessels. All liquid culture media were pre-reduced anaerobically at 37 °C for at least 1 h prior to use.

An optimised phage cocktail containing four *C. difficile* myoviruses, CDHM1, 2, 5, and 6 was used for phage therapy in this study. The individual phages were isolated from the environment and characterised previously in our laboratory [45,46], and propagated individually in liquid cultures of the environmental isolate CD105HE1 to produce 10^10^ PFU/mL of infective phage particles [45,53]. Prior to use, the phages were diluted to 10^9^ PFU/mL in BHI and mixed in equal proportions to constitute the cocktail, which was kept at 4 °C for short-term storage or in 25% glycerol for long-term storage at –80 °C.

### 4.2. Gut Fermentation Model Set-Up 

The gut fermentation model examined here was adapted from previous *C. difficile* phage studies [51] with slight modifications. The fermentation vessels were comprised of five 250 mL capacity Duran bottles containing 135 mL of a minimal medium containing 0.2% peptone, yeast extract, NaHCO_2_, and Tween 80, 0.01% NaCl, 0.004% each of K_2_HPO_4_ and KHPO_4_, 0.001% of MgSO_4_·7H_2_O, CaCl·2H_2_O, and vitamin K (in 5% aqueous solution), 0.005% each of Cysteine HCl and bile salts, and 0.0002% haemin (dissolved in 400 µL of 1 M NaOH). The pH of the medium was adjusted to and maintained at ~6.8 throughout the experiment, using filter sterilised NaOH and HCl. The medium was pre-reduced anaerobically at 37 °C for 24 h before use.

Freshly voided fecal samples were collected from four donors comprising a heathy White infant British boy (7 years old), a Black African teenage girl (17 years old), a Black African adult lady (44 years old) and a White British Elderly lady (70 years old). All donors were healthy at the time of sample collection and had not had antibiotics for 6 months prior to the time of sampling [56]. The fecal matter was passed at will into sterile plastic bowls before being transferred to Elkay 30 mL polystyrene transport tubes fortified with spoons. The samples were immediately stored under ice, and analysed within 2 h of collection. Under anaerobic conditions, approximately 5 g (approximately one spoonful within the Elkay tube) of each stool sample was diluted in 20 mL of the minimal media and mixed by inversion until all fecal materials were completely suspended to form a fecal emulsion or slurry.

One milliliter of each sample slurry was taken to determine the donors’ contributory microbiota. To do this, the culturable bacteria present in each fecal sample were ascertained by a viability assay on selective media for *C. difficile* (Brazier’s CCEY medium, BioConnections, Knypersley, UK), bifidobacteria (BSM medium, Sigma, Steinheim, Germany), total anaerobe (Wilkins-Chalgren anaerobic agar, Oxoid, Hampshire UK), lactobaccilli (Rogosa agar, Oxoid, Hampshire, UK), enterobacteria (MacConkey agar, Sigma, Steinheim, Germany), and Gram-positive cocci (Slanetz-Bartley agar, Oxoid, Hampshire, UK), prepared according to the manufactures’ recommendations. Afterwards, equal volumes of the fecal slurries were thoroughly mixed together, and 15 mL of the combined slurries was added to each of the fermentation vessels and further pre-reduced (anaerobically at 37 °C) for 2 h (−2 h, Table 1). The fermentation vessels were continuously agitated throughout the duration of the experiment using a sterile magnetic stirrer set, at 100 rpm. 

### 4.3. Bacteria and Phage Treatment of the Fermenation Vessels 

The five fermentation vessels were treated with 2 mL each of 6–8 × 10^8^ CFU/mL culture of the test strain CD105LC2 (B), 2–6 × 10^9^ PFU/mL of phage cocktail (P), and/or the minimal medium (M) at the time points shown in Table 1. At each time point, a 2 mL sample was removed from each of the treatment vessels and replaced with an equal volume of either the phage, the bacteria, or the minimum media, as appropriate (Table 1). The 2 mL samples were used for bacterial enumeration and DNA extraction for that time point. Vessel 1 (control untreated) contained the minimal medium and the fecal slurry only, and, at time points 0, 5, 24, 36, and 48 h, 2 mL of the pre-reduced minimal medium was added. In vessel 2 (*C. difficile* control), the bacterial culture was added at time point 0 h, and subsequently at the 5, 24, and 36 h time points, and at 48 h 2 mL of the minimal medium was added. For vessel 3 (control phage), the phages were added at 0, 5, 24, and 36 h, and at 48 h 2 mL of the medium was added. In the prophylactic regimen (vessel 4), the phages were added during the pre-reduction time (−2 h, on the basis of our prior data on phage pre-treatment time during the prophylaxis regimen [46]) and followed by the mixture of the phage and bacterial inocula, added at the 0 h time point. Afterwards, the phages were added at 5 and 24 h, and the medium at 36 and 48 h in vessel 4. For the remedial regimen (vessel 5), the bacterial inoculum was added at the 0 h time point, followed by the phage cocktail at the 5, 24, 36, and 48 h time points. The experiment was terminated at 72 h, and at this time the bacterial numbers were enumerated as described above.

### 4.4. DNA Extraction, Metagenomics Sequencing, and Analysis 

The DNA was extracted from 1 mL of the 2 mL aliquot taken from the treated vessels using FastDNA spin kit for feces (MP Biomedicals, Santa Ana, CA, USA). The DNA quality was ascertained using Nanodrop One (Thermo Fisher Scientific, Madison, WI, USA) and Qubit ds DNA HS Assay kit with Qubit 3 fluorometer (Invitrogen, Carlsbad, CA, USA). Total genomic DNA was prepared using the NexteraXT DNA sample preparation kit (Illumina, San Diego, CA, USA). Sequencing was performed on the MiSeq platform using V2 (2 × 250 bp) chemistry. The resulting fastq files were trimmed with Sickle using default parameters, and metagenomes were assembled using megahit. An overview of sample diversity for each metagenome was obtained using Kraken [72] and visualised using Pavian [73]. The reads were mapped against the genomes of interest using BWA-MEM [74]).

## 5. Conclusions

Taken together, our data showed that the phage cocktail examined specifically cleared *C. difficile* in both prophylactic and remedial regimens despite the competitive pressure imposed by the diverse human microbiota. Furthermore, a therapy using the optimised phages altered the human commensal bacteria such that specific bacterial groups associated with a healthy gut microbiota dominated. In summary, the use of phages removed the target pathogen and favorably modified the model gut microbiome. Both of these outcomes would be beneficial if the phages were to be used therapeutically, so our data supports their further development as therapeutic agents.

## Figures and Tables

**Table 1:** Bacteria and phage treatment regimens for the gut fermentation vessels.

Fermentation Vessels	Treatments	Time to Dose (h)						
		−2	0	5	24	36	48	72
1	Control untreated	-	M	M	M	M	M	-
2	*C. difficile* control	-	B	M	M	M	M	-
3	Control phage	-	P	P	P	P	M	-
4	Prophylaxis	P	P+B	P	P	M	M	-
5	Remedial	-	B	P	P	P	P	-

Five vessels containing combined fecal slurries from four healthy volunteers were treated with 2 mL each of 6–8 × 10^8^ CFU/mL of *C. difficile* culture (B), 2–6 × 10^9^ PFU/mL (plaque-forming units per milliliter) of phage cocktail (P), and/or minimal medium (M) at the time points shown above [51]. At each time point, 2 mL of samples from the vessels were removed and replaced with an equal volume of the bacteria, phage or medium. The time points were selected based on our prior in vitro data on the phages, which showed that the phages maintained clearance of CD105LC2 cultures at the first 5 and 24 h time points [45]. The additional 36 and 48 h time points were based on previous fermentation studies [51].

**Table 2:** Reads mapped to the three domains of life.

Domain	Clade Reads in Each Vessel (%)				
	V1	V2	V3	V4	V5
Bacteria	89,526 (97.76)	74,116 (99.05)	23,061 (98.89)	96,888 (98.77)	103,406 (99.11)
Archaea	2030 (2.217)	646 (0.8633)	19 (0.08148)	926 (0.944)	764 (0.7323)
Viruses	18 (0.01966)	64 (0.08553)	240 (1.029)	284 (0.2895)	165 (0.1581)
Total	91,574	74,826	23,320	98,098	104,335

Whole genome sequencing was conducted on DNA samples extracted at the 24 h time point. The data was analysed using Pavian.

## Supplementary Materials

The following are available online at http://www.mdpi.com/2079-6382/7/1/13/s1, Table S1: Percent bacterial families analysed from the metagenomics data using Pavian.

## Patents

The phages described are part of a Leicester patent, pending. European Patent Application No 13759275.4 and US Patent Application No. 14/423284.
