# Supplementary material for: Efficacy of an Optimised Bacteriophage Cocktail to Clear Clostridium difficile in a Batch Fermentation Model
Source: Antibiotics (Basel). 2018 Feb 13;7(1):13. doi: 10.3390/antibiotics7010013 (PMC5872124; doi:10.3390/antibiotics7010013)
Supplement: Supplementary file 1 [file antibiotics-07-00013-s001.pdf]

name		taxID	Max.1	%V1.cladeReads..	%V2.cladeReads..	%V3.cladeReads..	%V4.cladeRea	%V5.cladeReads..	lineage
Firmicutes	P	1239	47.81	41.62	37.57	25.84	47.81	45.75	cellular organisms>Bacteria
Bacteroidetes	P	976	31.41	30.32	31.41	23.31	24.91	26.21	cellular organisms>Bacteria>Bacteroidetes/Chlorobi group
Proteobacteria	P	1224	29.86	10.42	14.64	29.86	12.33	12.49	cellular organisms>Bacteria
Actinobacteria	P	201174	13.56	13.21	13.56	11.38	12.04	12.76	cellular organisms>Bacteria
Cyanobacteria	P	1117	8.761	0.1308	0.1063	8.761	0.1319	0.1711	cellular organisms>Bacteria
Euryarchaeota	P	28890	2.223	2.223	0.8629	0.0785	0.9436	0.7242	cellular organisms>Archaea
Verrucomicrobia	P	74201	1.36	1.36	1.152	0.1308	1.029	1.143	cellular organisms>Bacteria>Chlamydiae/Verrucomicrobia group
Deinococcus-Thermus	P	1297	0.1968	0.1352	0.144	0.1744	0.1968	0.1711	cellular organisms>Bacteria
Spirochaetes	P	203691	0.1669	0.1494	0.1319	0.1396	0.1669	0.1605	cellular organisms>Bacteria
Synergistetes	P	508458	0.09374	0.07253	0.07942	0.07414	0.09374	0.07445	cellular organisms>Bacteria
Chlorobi	P	1090	0.06864	0.06044	0.05654	0.03925	0.05254	0.06864	cellular organisms>Bacteria>Bacteroidetes/Chlorobi group
Acidobacteria	P	57723	0.06381	0.04286	0.03635	0.05669	0.05872	0.06381	cellular organisms>Bacteria>Fibrobacteres/Acidobacteria group
Chloroflexi	P	200795	0.06327	0.04176	0.06327	0.05233	0.05666	0.05414	cellular organisms>Bacteria
Fusobacteria	P	32066	0.04286	0.04286	0.02692	0.01308	0.02781	0.02127	cellular organisms>Bacteria
Planctomycetes	P	203682	0.04018	0.02857	0.02423	0.03053	0.04018	0.02707	cellular organisms>Bacteria
Tenericutes	P	544448	0.03769	0.03516	0.03769	0.008722	0.02575	0.02224	cellular organisms>Bacteria
Thermotogae	P	200918	0.02019	0.01099	0.02019	0.004361	0.01545	0.01064	cellular organisms>Bacteria
Chrysiogenetes	P	200938	0.01545	0.01538	0.008077	NA	0.01545	0.008701	cellular organisms>Bacteria
Fibrobacteres	P	65842	0.01308	0.008791	0.009423	0.01308	0.01236	0.01064	cellular organisms>Bacteria>Fibrobacteres/Acidobacteria group
Gemmatimonadetes	P	142182	0.01212	0.00989	0.01212	0.008722	0.008241	0.009668	cellular organisms>Bacteria
Chlamydiae	P	204428	0.01209	0.01209	0.006731	NA	0.007211	0.005801	cellular organisms>Bacteria>Chlamydiae/Verrucomicrobia group
Crenarchaeota	P	28889	0.0116	0.006593	0.006731	0.004361	0.008241	0.0116	cellular organisms>Archaea
Nitrospirae	P	40117	0.01064	0.00989	0.006731	NA	0.007211	0.01064	cellular organisms>Bacteria
Elusimicrobia	P	74152	0.009271	0.005494	0.004039	0.004361	0.009271	0.003867	cellular organisms>Bacteria
Deferribacteres	P	200930	0.006593	0.006593	0.005385	NA	0.00206	0.005801	cellular organisms>Bacteria
Aquificae	P	200783	0.005801	0.004395	0.004039	0.004361	0.005151	0.005801	cellular organisms>Bacteria
Ignavibacteriae	P	1134404	0.004395	0.004395	NA	NA	0.00206	0.003867	cellular organisms>Bacteria>Bacteroidetes/Chlorobi group
Armatimonadetes	P	67819	0.004361	0.002198	0.004039	0.004361	0.00103	0.0009668	cellular organisms>Bacteria
Thermodesulfobacteria	P	200940	0.003297	0.003297	NA	NA	0.00103	NA	cellular organisms>Bacteria
Cloacimonetes	P	456828	0.002692	0.002198	0.002692	NA	NA	0.001934	cellular organisms>Bacteria>unclassified Bacteria
Caldiserica	P	67814	0.002692	0.001099	0.002692	NA	0.00206	0.0009668	cellular organisms>Bacteria
Dictyoglomi	P	68297	0.00206	0.001099	0.001346	NA	0.00206	0.0009668	cellular organisms>Bacteria
Candidatus Saccharibacteria	P	95818	0.001346	0.001099	0.001346	NA	NA	NA	cellular organisms>Bacteria>unclassified Bacteria
Thaumarchaeota	P	651137	0.001099	0.001099	NA	NA	NA	0.0009668	cellular organisms>Archaea
